# Organic Solvent Nanofiltration Membrane with In Situ Constructed Covalent Organic Frameworks as Separation Layer

**DOI:** 10.3390/membranes14110234

**Published:** 2024-11-08

**Authors:** Fangyi Xu, Shuxin Zhao, Junjie Song, Yu Peng, Baowei Su

**Affiliations:** 1 Key Laboratory of Marine Chemistry Theory and Technology, Ministry of Education, Ocean University of China, 238 Songling Road, Qingdao 266100, China; xfy_ouc@126.com (F.X.); zhaoshuxin_ouc@126.com (S.Z.); sjj_ouc@126.com (J.S.); 2 College of Chemistry & Chemical Engineering, Ocean University of China, 238 Songling Road, Qingdao 266100, China

**Keywords:** organic solvent nanofiltration (OSN), covalent organic frameworks (COFs), interfacial polymerization (IP), in situ construction

## Abstract

Organic solvent nanofiltration (OSN) technology is advantageous for separating mixtures of organic solutions owing to its low energy consumption and environmental friendliness. Covalent organic frameworks (COFs) are good candidates for enhancing the efficiency of solvent transport and ensuring precise molecular sieving of OSN membranes. In this study, p-phenylenediamine (Pa) and 1,3,5-trimethoxybenzene (Tp) are used to construct, in situ, a TpPa COF skin layer via interfacial polymerization (IP) on a polyimide substrate surface. After subsequent crosslinking and activation steps, a kind of TpPa/polyimide (PI) OSN membrane is obtained. Under optimized fabrications, this OSN membrane exhibits an ethanol permeance of 58.0 LMH/MPa, a fast green FCF (FGF) rejection of 96.2%, as well as a pure *n*-hexane permeance of 102.0 LMH/MPa. Furthermore, the TpPa/PI OSN membrane exhibits good solvent resistance, which makes it suitable for the separation, purification, and concentration of organic solvents.

## 1. Introduction

Organic solvent nanofiltration (OSN) separation technology is advantageous over traditional distillation methods owing to its low energy consumption and high selectivity [1]. OSN membranes exert a direct influence on the efficiency of the OSN separation process. To date, researchers have prepared a variety of OSN membranes with pore sizes ranging from several nanometers to sub-nanometers. However, conventional polymer materials generally lack tunable and well-organized pore channels, leading to limited membrane porosity and uneven pore distribution, thus affecting the separation performance of OSN membranes.

Covalent organic frameworks (COFs) are suitable for the fabrication of membranes for rapid molecule transport and precise molecular sieving due to their porous crystalline structure [2,3]. The use of COFs in membrane preparation could be classified into three main categories: doping the synthesized COFs as additives into the monomer solutions during the interfacial polymerization (IP) process for membrane preparation [4,5], using COFs to construct an interlayer [5], or directly using COFs to construct the separation layer [6,7]. Among them, membranes with COFs as the separation layer have drawn more attention with the advancements in COF synthesis technology.

COF membranes have promising applications in gas separation [8], water treatment [9], pervaporation [10], and organic solvent nanofiltration [11,12]. Until now, most of the research works have been done on nanofiltration (NF) membranes. For instance, Kandambeth et al. [12] showcased an eloquent fabrication methodology for the production of a series of self-standing, porous, and crystalline COF membranes. They utilized these membranes for the recovery of valuable active pharmaceutical ingredients (APIs) from organic solvents as the representative cases. Dey et al. [13] prepared a Tp-Bpy self-supporting COF NF membrane via a 72 h reaction between 5,5′5′-diamino-2,2′2′-bipyridine (Bpy) and 1,3,5-tricarbonylresorcinol (Tp). The Tp-Bpy membrane exhibited an acidic fuchsin (AF) rejection rate of 97% and an exceptionally high pure water permeance of 2110 LMH/MPa. However, the extremely long reaction time of the self-supporting COF membrane greatly limits its application. In order to facilitate the practical application of COF membranes, Wang et al. [14] fabricated COF NF membranes using Tp and p-phenylenediamine (Pa) as reaction monomers via IP on polysulfone (PSf) substrates. Due to the moderate reaction rate between monomer pairs in aqueous and organic solutions, a COF can be conformally grown in less than 1 min. The prepared membrane exhibited 99.5% congo red (CR) rejection rate and a pure water permeance of 500 LMH/MPa. The above results demonstrate that the high-performance COF NF membrane can be prepared via IP in a very short time. In addition, in response to the still existing problem of uneven and discontinuous COF structures caused by an extremely short reaction time, Lei et al. [15] used a very low concentration (0.6 mM) of Tp with the Pa monomer to pre-generate oligomers with a planar structure in *n*-hexane, and then catalyzed the oligomers by acetic acid for 60 s on a PSf-based membrane surface. Afterward, they cured the membrane at 80 °C for 5 min and achieved a pure water permeance of 652 LMH/MPa as well as a CR rejection rate of 98.0%.

Currently, most membranes with COFs as the separation layer are used for aqueous systems, and relatively few are used for organic solvent systems. Nevertheless, COFs have an excellent resistance to most organic solvents, and are promising candidates for fabricating OSN membranes. Therefore, in this work, we construct an integrally crosslinked COF membrane via an IP process using Tp and Pa monomers on a polyimide (PI) substrate membrane surface within a short time, and, subsequently, perform crosslinking and solvent activation. The optimized TpPa/PI COF membrane exhibits effective separation performance and remarkable resistance to strong polar solvents. This work provides insights into the fabrication of COF OSN membranes.

## 2. Materials and Methods

### 2.1. Materials

The materials used in this work are detailed in Table S1.

### 2.2. Fabrication Process of the TpPa/PI Membrane

**Figure 1:**
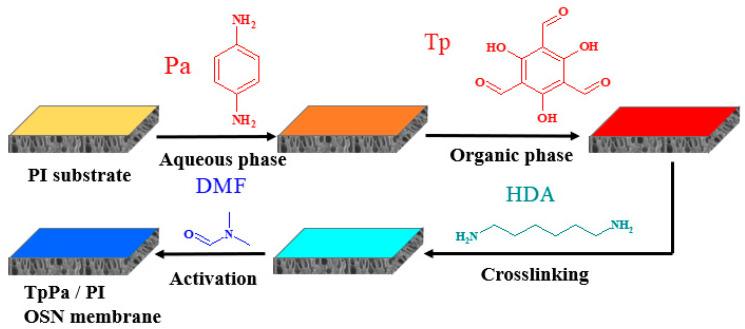
Fabrication process of the TpPa/PI membrane.

Prior to the fabrication of the TpPa/PI membrane, the PI substrate was prepared following the steps outlined in our previous work [16]. Afterward, the TpPa separation layer was constructed via the IP process, during which the aqueous phase solution (25 mL) containing a specific quantity of Pa, cetrimonium bromide (CTAB), 1 wt% acetic acid, and deionized water was poured onto the PI substrate surface (approximately 8 × 8 cm), where it remained for 30 s. Then, the aqueous phase solution was drained off and the organic phase solution (20 mL) containing Tp and *n*-hexane was poured thereon to perform the IP reaction and form the COF layer. The membrane was immediately heat cured at 60 °C for a certain time, then it was immersed in a 10 wt% 1,6-hexanediamine (HDA)/isopropanol (IPA) solution at 60 °C for 30 min to facilitate the crosslinking. Afterward, it was immersed in ethanol to replace the crosslinking agent, then it was immersed in N, N-dimethylformamide (DMF) at 80 °C for 15 min to perform the activation. The fabrication process is shown in Figure 1.

### 2.3. Characterization Methods

The characterization methods used of the membrane are detailed in Table S3.

### 2.4. Separation Performance Test

All the separation tests, including the long-term filtration test, were performed using our crossflow filtration platform at 0.5 MPa and 25 °C, with a test area of 28.26 cm^2^. The permeance (*P*) and the rejection (*R*) were calculated according to Equations (S1) and (S2), respectively, in the Supplementary Material.

During the separation performance test, 100 mg L^−1^ of Fast Green FCF (FGF, 809 Da)/ethanol solution was used as feed. The pure solvent permeance was also tested using different pure organic solvents, of which the characteristic parameters are presented in Table S4. Further, a kind of solution with a weakly polar solvent, 100 mg L^−1^ Jacobsen catalyst/ethyl acetate mixture, was also selected as feed to evaluate the potential application of the COF membrane in chemical industries.

### 2.5. MWCO of the Membrane

The ethanol solutions consisting of 100 mg L^−1^ safranine T (ST), rhodamine B (RDB), eosin Y (EY), and FGF were used to evaluate the molecular weight cut-off (MWCO) of the TpPa/PI membrane.

### 2.6. Long-Term Solvent Resistance Test

The TpPa/PI membranes were immersed in 25 °C DMF solution for 23 days, and the performance was evaluated using 100 mg L^−1^ of FGF/ethanol solution as feed at 0.5 MPa and 25 °C to evaluate the solvent resistance.

## 3. Results and Discussion

### 3.1. Characterization of the Membrane

#### 3.1.1. FTIR

**Figure 2:**
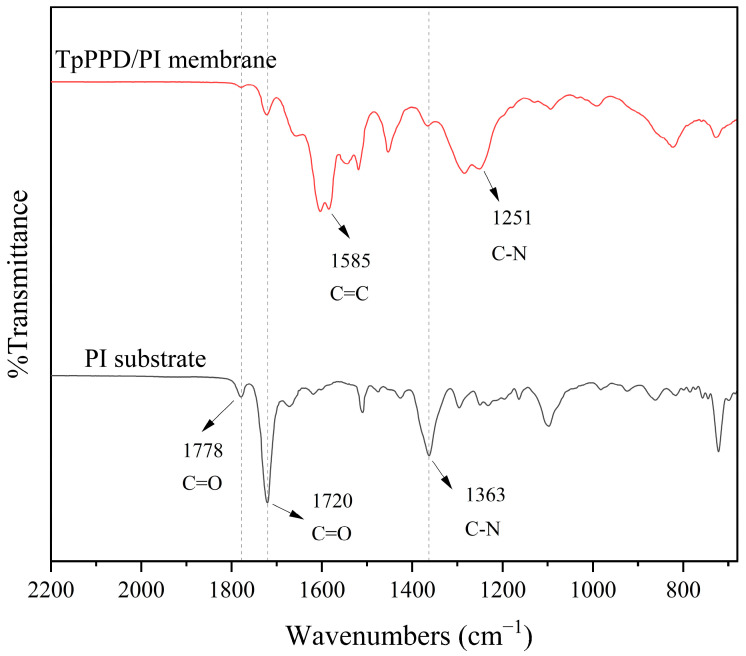
FTIR spectra of the membranes.

**Figure 3:**
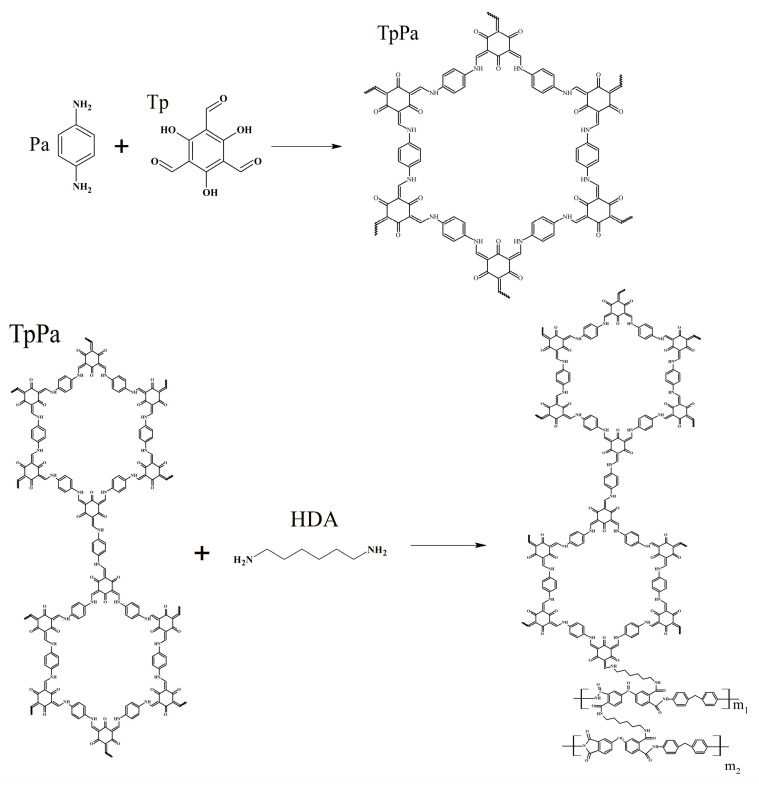
Possible reaction processes.

As shown in Figure 2, the peaks at 1251 cm^−1^ and 1585 cm^−1^ corresponded to the stretching vibration peak of the C-N and C = C bonds in the TpPa COF layer [14], which confirm the successful construction of the TpPa COF separation layer. Figure 3 illustrates the possible reactions during this process.

#### 3.1.2. XRD Characterization

**Figure 4:**
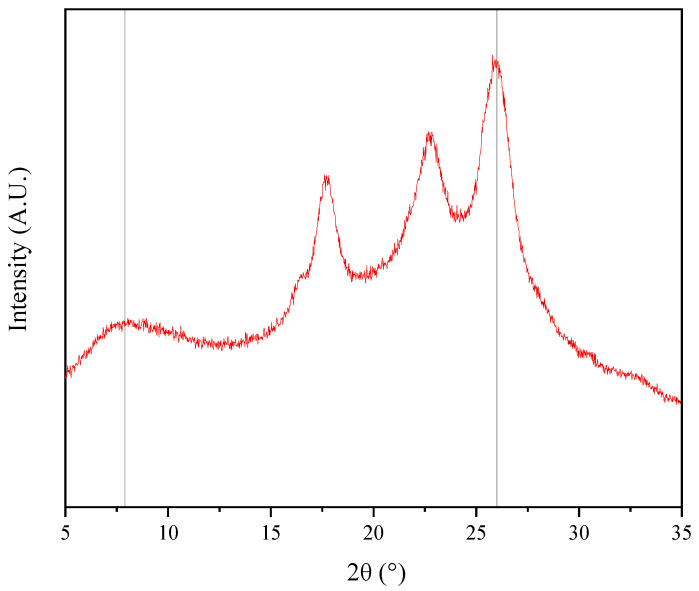
XRD pattern of the TpPa/PI membrane.

As shown in Figure 4, the TpPa/PI composite membrane had characteristic peaks at 2θ = 7.9° and 26.0°. The peak at 2θ = 7.9° represents the characteristic peak of the TpPa COF material [14], proving the successful synthesis of the TpPa COF. The peak at 2θ = 26.0° represents the peak generated by the π-π stacking of benzene rings [17], since the growth of the TpPa COF in space is the stacking of two-dimensional network COFs, a phenomenon which could generate the π-π stacking of benzene rings.

#### 3.1.3. Surface Topography Characterization

**Figure 5:**
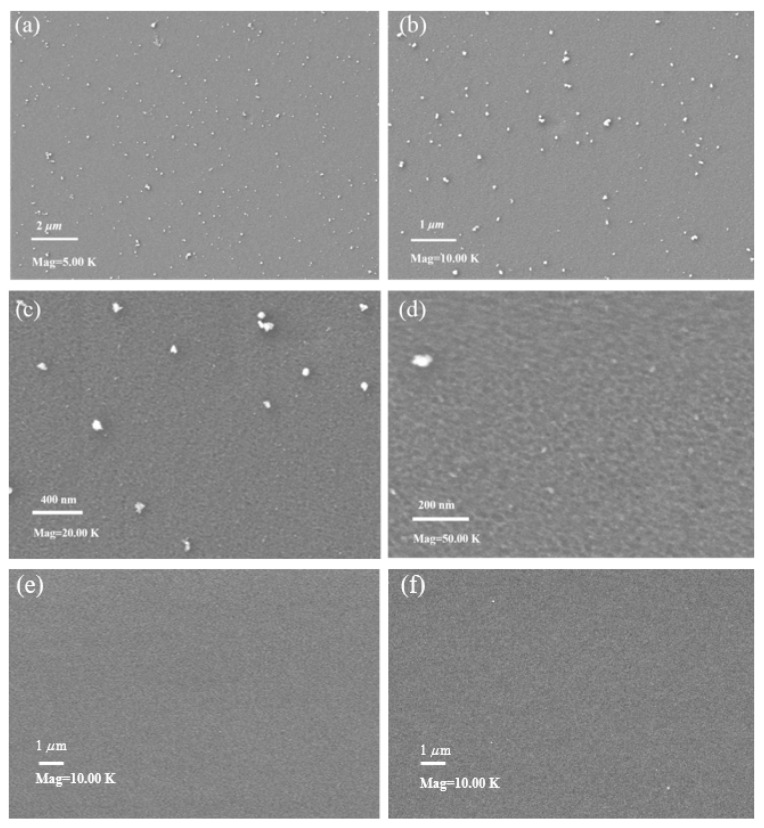
SEM images of the TpPa/PI composite membrane (**a**) magnification = 5 K, (**b**) magnification = 10 K, (**c**) magnification = 20 K, (**d**) magnification = 50 K; PI substrate (**e**) magnification = 10 K; PI substrate after crosslinking and activation steps (**f**) magnification = 10 K.

As shown in Figure 5, the surface of the TpPa/PI membrane in Figure 5a–d clearly showed COF structures compared to the PI substrate and the PI substrate after the crosslinking and activation steps in Figure 5e,f, indicating that the TpPa COF separation layer had been experimentally synthesized. The surface of the TpPa/PI membrane was flat, which is due to the fact that the TpPa COF material is a two-dimensional monolayer structure when it grows spatially [18]. This uniform and flat surface is beneficial for reducing the deposition of contaminants thereon, i.e., it is beneficial for improving the fouling resistance of the membrane [19].

**Figure 6:**
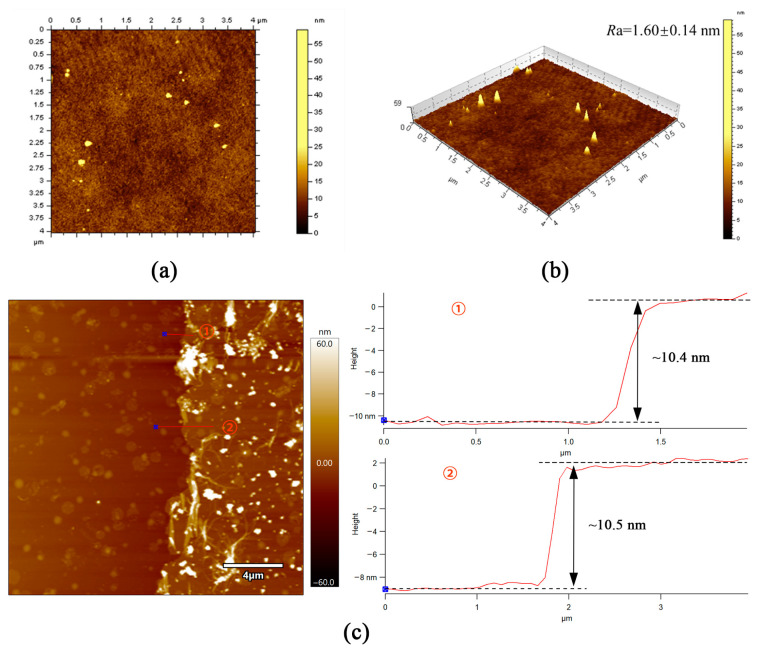
(**a**,**b**) AFM images and surface roughness of the TpPa/PI membrane; (**c**) AFM and height profiles of the self-standing COFs layer.

In Figure 6a,b, the average surface roughness (*R*a) of the TpPa/PI composite membrane is as low as 1.6 nm, which is consistent with the SEM results. Further, it is much lower than most of the other OSN membranes in the literature [20,21,22], as shown in Table S5. As shown in Figure 6c, the thickness of the COF layer via AFM using a self-standing film fabricated under the same concentration of COF monomers was as thin as about 10.5 nm.

#### 3.1.4. Hydrophilicity Characterization

The TpPa/PI membrane in this work and the PI substrate and other COF OSN membranes in the literature are shown in Table 1. The TpPa/PI composite membrane had a WCA value of 78.8°, which is much higher than that of the PI substrate in our previous work. The construction of the TpPa COF layer effectively enhanced the hydrophobicity of the membrane surface, a phenomenon which is suitable for transmembrane transport of weakly polar solvents and non-polar solvents.

### 3.2. Effect of the Fabrication Condition

#### 3.2.1. Monomers Concentration

**Figure 7:**
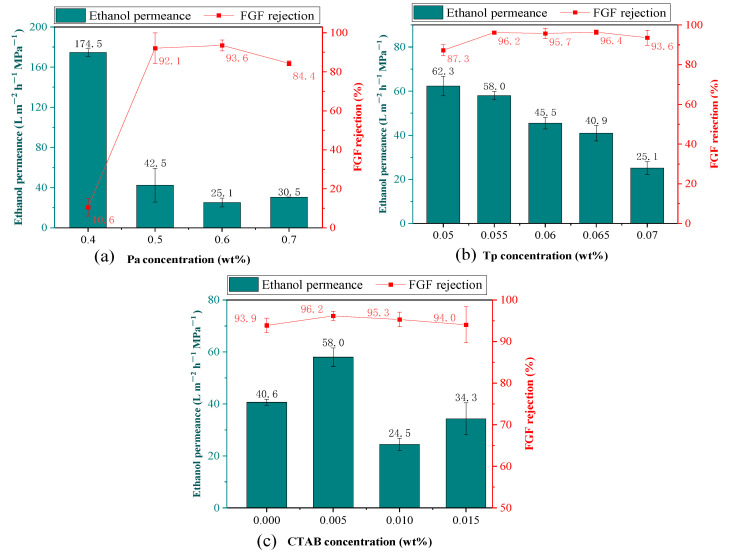
Effect of the concentration of (**a**) Pa, (**b**) Tp, and (**c**) CTAB on the separation performance of the TpPa/PI membrane using the fabrication of (**a**) Tp, concentration: 0.070 wt%, and CTAB, concentration: 0.005 wt%, (**b**) Pa, concentration: 0.6 wt%, and CTAB, concentration: 0.005 wt%, (**c**) Pa, concentration: 0.6 wt%, and Tp concentration: 0.055 wt%. Other conditions included IP time: 40 s, heat treatment time: 5 min, and solvent activation time: 15 min.

As shown in Figure 7a, the FGF rejection rate increased sharply from 10.6% to 93.6% as the Pa concentration increased from 0.4 wt% to 0.6 wt%, accompanied by a sharp decrease in ethanol permeance from 174.5 LMH/MPa to 25.1 LMH/MPa. This could be due to the fact that the constructed COF layer became increasingly homogeneous, complete, and less defective with the increase in Pa concentration, thus leading to the increase of the rejection rate and to the decrease of the permeance [19]. As the Pa concentration further increased to 0.7 wt%, there was a slight increase in the ethanol permeance, while the FGF rejection rate decreased from 93.6% to 84.4%. This could be due to the acceleration of the IP reaction rate caused by the increased Pa concentration. This, in turn, resulted in a decrease in the crystallinity of the COF layer, thus leading to an increase in the defects of the COF layer [13]. Consequently, a 0.6 wt% Pa solution was selected for further analysis.

As shown in Figure 7b, the ethanol permeance decreased gradually from 62.3 LMH/MPa to 25.1 LMH/MPa as the Tp concentration increased from 0.05 wt% to 0.07 wt%. This suggests that the hydraulic resistance became higher with the increase in Tp concentration, a phenomenon which might be due to the gradually thicker skin layer that was detrimental to solvent rapid transport [14]. The FGF rejection rate increased as the Tp concentration further increased from 0.05 wt% to 0.065 wt%, suggesting that the increase in Tp concentration effectively reduced the defects of the TpPa COF layer [14]. The further increased Tp concentration from 0.065 wt% to 0.070 wt% resulted in a slight decline in the FGF rejection rate from 96.2% to 93.6%, which is due to the low Tp solubility in *n*-hexane solution, thus leading to the agglomeration of the Tp monomers and the uneven IP reaction and resulting in the formation of defects in the TpPa COF layer and the decrease in the FGF rejection rate. Consequently, a 0.055 wt% Tp solution was selected for further analysis.

As shown in Figure 7c, the FGF rejection rate increased from 93.9% to 96.2% as the CTAB concentration increased from 0% to 0.005 wt%, since CTAB could cause a more uniform diffusion of the aqueous monomer at the IP interface, thus resulting in a more complete COF layer and in an increase in the FGF rejection rate. Moreover, the ethanol permeance increased from 40.6 LMH/MPa to 58.0 LMH/MPa. However, the FGF rejection rate decreased from 96.2% to 93.9% as the CTAB concentration increased from 0.005 wt% to 0.015 wt%. The ethanol permeance firstly decreased from 58.0 LMH/MPa to 24.5 LMH/MPa, then increased to 34.3 m^−2^ h^−1^ MPa^−1^. As a surfactant, CTAB could facilitate the diffusion of amine monomers at the phase interface in a more rapid and homogeneous manner [24], resulting in the formation of a separation layer with uniform sub-nanopores [24,25] which increases the solute rejection rate and decreases the solvent permeance. The further increased CTAB concentration increased the diffusion rate of the aqueous phase monomer into the organic phase, thus resulting in a high concentration of the aqueous phase monomer in the IP interface and a drastic and rapid IP reaction. In this case, it is difficult for a thin, continuous, and dense separation layer to form. This, in turn, leads to many defects and a high thickness of the separation layer as well as a decrease in the FGF rejection rate and the ethanol permeance [25]. Consequently, a 0.005 wt% CATB solution was selected for the follow-up study.

#### 3.2.2. Effect of IP Reaction Time

**Figure 8:**
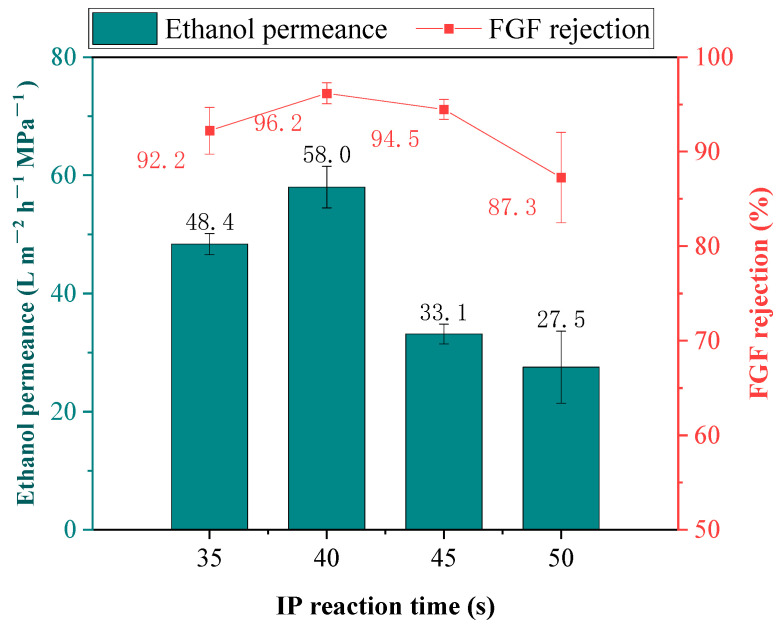
Effect of IP reaction time on the separation performance of the TpPa/PI membrane (other conditions: Pa concentration: 0.6 wt%, Tp concentration: 0.055 wt%, CTAB concentration: 0.005 wt%, heat treatment time: 5 min).

As illustrated in Figure 8, the increase in IP time from 35 s to 40 s resulted in an increase in the FGF rejection rate from 92.2% to 96.2% and in an increase in the ethanol permeance from 48.4 LMH/MPa to 58.0 LMH/MPa. The increased FGF rejection suggests that the slightly longer reaction time (40 s) resulted in significantly fewer defects in the COF separation layer. As a result, during the filtration of the dye solution, fewer dye molecules could be adsorbed and trapped in the pores of the COF separation layer, thus exerting less resistance against solvent transport [26]. Therefore, the ethanol permeance of the OSN membrane fabricated at a reaction time of 40 s was higher than that of the membrane fabricated at a reaction time of 35 s. However, with the IP time further increasing from 40 s to 50 s, the FGF rejection rate decreased from 96.2% to 87.3% and the ethanol permeance decreased from 58.0 LMH/MPa to 27.5 LMH/MPa. This suggests that the increased IP time led to a thicker TpPa COF separation layer with a higher hydraulic resistance. On the other hand, the increased IP time also decreased the number of residual aqueous monomer molecules. Thus, fewer residual aqueous monomer molecules could react with the organic phase monomer in the subsequent heat treatment process, making it difficult to fill up the defects of the separation layer during this curing process and leading to a decrease in FGF rejection rate. Therefore, a short IP reaction time of 40 s was selected in this work. Usually, the formation of a COF separation layer often requires a long reaction time of several hours or even days [13,27,28], while our work achieved a very high dye rejection rate in a very short reaction time. The same result was also seen in the work by Prof. Yong Wang [14] in which the COF membrane prepared in their work achieved a CR rejection rate of 99.5% and a water permeance of 500 LMH/MPa or 50 ppm CR/H_2_O solution in a reaction time of 10 s.

### 3.3. Separation Performance of the Membrane

#### 3.3.1. Pure Solvent Permeance

**Figure 9:**
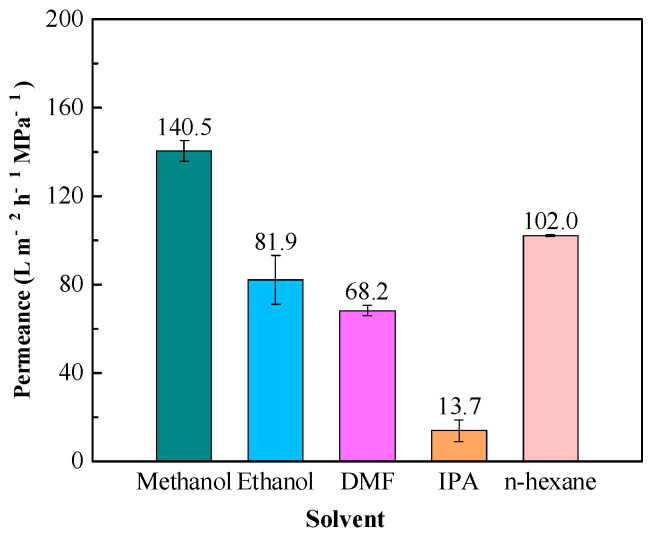
Pure solvent permeance of the TpPa/PI membrane.

In Figure 9, the TpPa/PI OSN membrane has a high methanol permeance of 140.5 LMH/MPa because of its smallest molecular volume. The permeances for ethanol, DMF, and isopropanol were 81.9 LMH/MPa, 13.7 LMH/MPa, and 68.2 LMH/MPa, respectively. It is worth noting that the TpPa/PI membrane had a high *n*-hexane permeance of 102.0 LMH/MPa, since the TpPa/PI membranes had a higher surface hydrophobicity, as discussed in Section 3.1.4, thus leading to a higher permeance of non-polar solvents.

#### 3.3.2. MWCO

**Figure 10:**
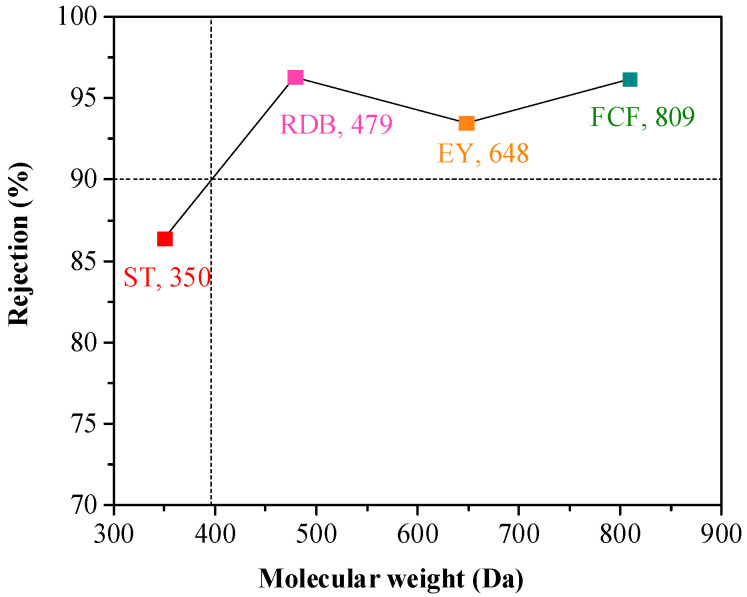
The rejection of the TpPa/PI membrane for dyes.

In Figure 10, the TpPa/PI membrane shows rejection rates higher than 93% for RDB, EY, and FGF, and a rejection rate lower than 87% for ST. It can be seen that the corresponding solvent molecular weight had a rejection rate of 90%, i.e., the MWCO was about 396 Da.

#### 3.3.3. For Weakly Polar Solvent System

Jacobson’s catalyst is an effective homogeneous catalyst that catalyzes epoxidation reactions and has become an important catalyst in the chemical industry owing to its high chemical selectivity [29]. However, in the homogeneous catalysis system, the efficient separation and recovery of catalysts is a great challenge [30]. In this study, the separation performance of the TpPa/PI COF OSN membrane fabricated in this work on the Jacobson’s catalyst in an ethyl acetate solvent solution was tested and compared with those of other OPN membranes in the literature [31,32,33], and the results are shown in Table 2. It can be seen that the TpPa/PI COF OSN membrane prepared in this study had a reasonably high rejection rate of 87% for the Jacobson’s catalyst and a superior permeance of 75.8 LMH/MPa for ethanol acetate, indicating its excellent permeation performance in relation to weakly polar solvents and proving its effectiveness in separating Jacobson’s catalysts in chemical industries.

#### 3.3.4. Long-Term Solvent Resistance

**Figure 11:**
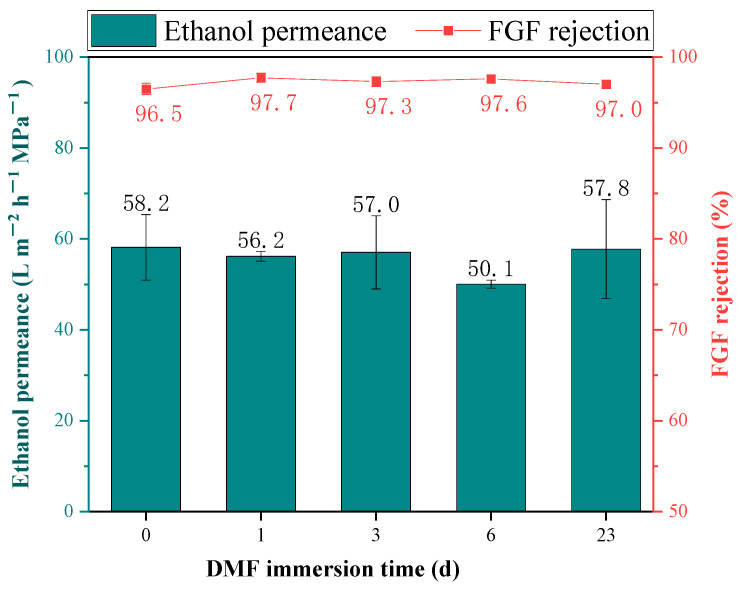
Long-term stability of the TpPa/PI membrane.

The results of the long-term solvent-resistant stability of the TpPa/PI membrane are illustrated in Figure 11. After a 25 °C DMF immersion of 23 days, the ethanol permeance of the TpPa/PI COF OSN membrane remained almost constant, and the FGF rejection rate slightly increased from 96.5% to 97.0%. The results demonstrate that the TpPa/PI membrane has outstanding solvent resistance against strong polar solvents.

#### 3.3.5. Benchmark

The separation performance of the COF OSN membrane fabricated in this work and that of other COF OSN membranes reported in the literature [34,35,36] is shown in Table 3. It can be seen that the TpPa/PI membrane prepared in this study has a relatively higher solute rejection rate than that of other OSN membranes. Although the Tp-Azo membrane has a higher solute rejection, it has a much longer reaction time. Further, the TpPa/PI membrane prepared in this study shows a much higher pure *n*-hexane permeance than that of other COF OSN membranes, indicating that the COF membrane we fabricated is also well suited for use in non-polar solvent systems.

## 4. Conclusions

In this study, we detail the generation of an OSN membrane which has an overall crosslinking structure with a COF layer as the separation layer. The prepared TpPa/PI membrane has a smooth surface and an average roughness of about 1.60 nm. The optimal TpPa/PI membrane exhibits a high permeance relative to both polar and non-polar solvents, with a methanol permeance of 156.7 LMH/MPa and a high *n*-hexane permeance of 102.0 LMH/MPa, while the *n*-hexane permeance is approximately one order of magnitude higher than that of most other OSN membranes reported in the literature (Table 3). In addition, the TpPa/PI membrane shows excellent solvent resistance, proving its high potential prospects for separation and purification for organic solvent systems.

## Figures and Tables

**Table 1:** WCA of the TpPa/PI membrane in this work and the PI substrate and other COF OSN membranes in the literature.

Membrane	WCA (°)	Literature
TpPa/PI membrane	78.8	This work
PI substrate	40.7	Our previous work [23]

**Table 2:** Separation performance of TpPa/PI membrane in relation to catalysts compared with those of other OSN membranes in the literature [31,32,33].

Membrane	Catalyst	Solvent	Solvent Permeance (LMH/MPa)	Catalyst Rejection (%)	Reference
TpPa/PI	Jacobson’s catalyst	Ethanol acetate	75.8	87.1%	This work
COK M2	Jacobson’s catalyst	Ethyl ether	2.2	83.0%	[31]
DuraMem 300	Magnesium trifluoromethanesulfonate	Ethanol/Ethyl acetate/Cyclohexane	1.0	98.0%	[32]
PERVAP 4060	Tetraoctylammonium bromide	Toluene	20.0	92.0%	[33]

**Table 3:** Comparison of the separation performance of the COF OSN membrane fabricated in this work and that of other COF OSN membranes reported in the literature.

Membrane	Reaction Time	Solute/Solvent	Permeance (LMH/MPa)	Rejection (%)	Pure *n*-Hexane Permeance (LMH/MPa)	Literature
TpPa/PI membrane	40 s	FGF/ethanol	58.0	96.2	102.0	This work
TpPaAn-30/HPaN membrane	30 s	Methyl blue/methanol	318	>90	67.0	[34]
COP-30 membrane	10 s	CR/toluene	111.0	>95	47.0	[35]
Tp-Azo membrane	72 h	Gold nanoclusters/acetonitrile	—	~100	—	[36]

## Data Availability

The data that support the findings of this study are available upon request from the corresponding author.

## Supplementary Materials

The following supporting information can be downloaded at: https://www.mdpi.com/article/10.3390/membranes14110234/s1, Table S1: Experimental materials; Table S2: The information of dyes used in this study; Table S3: Characterization of the membrane; Table S4: Physical properties of different solvents used in experiments; Table S5: Comparison of Ra between the TpPa/PI membrane and COF membranes reported in the literature.
