# Supplementary material for: Organic Solvent Nanofiltration Membrane with In Situ Constructed Covalent Organic Frameworks as Separation Layer
Source: Membranes (Basel). 2024 Nov 8;14(11):234. doi: 10.3390/membranes14110234 (PMC11596232; doi:10.3390/membranes14110234)
Supplement: Supplementary file 1 [file membranes-14-00234-s001.zip › membranes-3243170-supplementary.pdf]

Supplementary Material

Organic Solvent Nanofiltration Membrane with in Situ Constructed Covalent Organic Frameworks as Separation Layer

Fangyi Xu[a, b,1], Shuxin Zhao[a, b,1], Junjie Song[a,b], Yu Peng[a,b,*], Baowei Su[a,b,*]

[a] Key Laboratory of Marine Chemistry Theory and Technology (Ocean University of China), Ministry of Education, 238 Songling Road, Qingdao, 266100, China

[b] College of Chemistry & Chemical Engineering, Ocean University of China, 238 Songling Road, Qingdao, 266100, China

[1] First authors, Fangyi Xu and Shuxin Zhao contribute equally

[*] Corresponding authors, Email: py_ouc@126.com (Y. Peng)
subaowei@ouc.edu.cn (B. Su)

1. Experimental materials

Table S1 Experimental materials

Name	Specification	Manufacturers
Polyimide (Lenzing P84)	/	HP Polymer GmbH (Austria)
Polyester (PET) non-woven	/	Teijin Co., Ltd. (Japan)
p-Phenylenediamine (Pa)	99%	Shanghai Macklin Biochemical Co., Ltd
1,3,5-triformylphloroglucinol (Tp)	95%	Bide Pharmatech Co., Ltd.
Acetic acid (CH3COOH)	A.R.	Tianjin Fuyu Fine Chemical Co., Ltd
N, N-dimethylformamide (DMF)	A.R.	Sinopharm Chemical Reagent Co., Ltd
Cetyltrimethylammonium bromide (CTAB)	A.R.	Tianjin Guangfu Fine Chemical Research Institute
Methanol (MeOH)	A.R.	Sinopharm Chemical Reagent Co., Ltd
Anhydrous ethanol (EtOH)	A.R.	Sinopharm Chemical Reagent Co., Ltd

n-hexane	A.R.	Sinopharm Chemical Reagent Co., Ltd
Ethyl acetate (EtOAc)	A.R.	Tianjin Fuyu Fine Chemical Co., Ltd
Isopropanol (IPA)	A.R.	Tianjin Fuyu Fine Chemical Co., Ltd
1,6-Hexanediamine (HDA)	A.R.	Sinopharm Chemical Reagent Co., Ltd
Polyethylene glycol 400 (PEG-400)	A.R.	Sinopharm Chemical Reagent Co., Ltd
Fast Green FCF (FGF, 809 Da)	B. S	Shanghai Macklin Biochemical Co., Ltd
Rhodamine B (RDB, 479 Da)	B. S	Sinopharm Chemical Reagent Co., Ltd
Safranine T (ST, 350 Da)	B. S	Shanghai Macklin Biochemical Co., Ltd
Eosin Y (EY, 647 Da)	B. S	Nanjing Duly Biotech Co., Ltd
(R, R) -(-)-N, N'-Bis(3,5-di-tert-butylsalicylidene)-1,2-cyclohexanediaminomanganese (III) chloride (Jacobson's catalyst, 635 Da)	A.R.	Shanghai Macklin Biochemical Co., Ltd
Deionized water	/	FDY1002-UV-P

2. The information of dyes used in this study

Table S2 The information of dyes used in this study

Dye	Charge	Molecular weight (Da)	Structure	Size (Å)
Fast green FCF	Negative	809	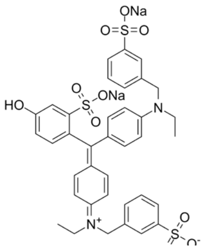	37.1×15.5×11.6
Rhodamine B	Positive	479	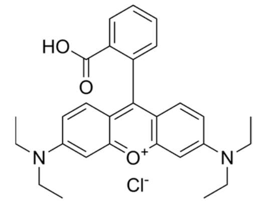	30.7×15.3×13.3
Safranine T	Positive	350	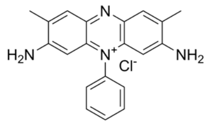	16.8×11.5×5.1
Eosin Y	neutral	648	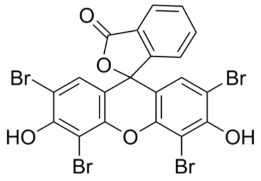	13.3×12.7×8.5

3. Characterization of the membrane

Table S3 Characterization of the membrane

Characterization techniques	Model	Company
Fourier transform infrared spectroscopy (FTIR)	Magna-560	Thermo Fisher Scientific
X-ray diffraction (XRD)	D8 ADVANCE diffractometer	Bruker Corporation
Atomic force microscopy (AFM)	Agilent-5400	Agilent Technologies Inc.
Scanning electron microscopy (SEM)	S-4800	Thermo Fisher Scientific
Contact angle (CA)	DSA-100	KRÜSS Scientific

4. Equations used in experiments

Permeate sample was collected as a function of time and was weighted by an electric balance to determine the solvent permeance (P, LMH/MPa) according to Eq. (1):

$$P = \frac{\Delta V}{A \cdot \Delta t \cdot \Delta p} \qquad Eq.(S1)$$

where ΔV (L) is the volume of the permeate collected in the time period Δt (h) under a trans-membrane pressure Δp (MPa), A (m^2) is the effective membrane area.

The solute (dye) rejection was calculated from Eq. (2):

$$R = \left(1 - \frac{C_p}{C_f}\right) \times 100\% \qquad Eq.(S2)$$

where C_p and C_f denote the dye concentrations in the permeate and in the feed solution, respectively. The dye concentration was measured by using an ultraviolet and visible spectrophotometer (METASH UV-5100, China).

5. Physical properties of different solvents used in experiments

Table S4 Physical properties of different solvents used in experiments

Solvent	Molecular weight (Da)	Molecular structural formula	Density (g cm^{-3})	Dielectric Constant (25 ℃)
DMF	73	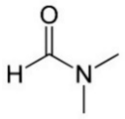	0.95	36.7
Methanol	32	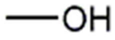	0.79	32.2
Ethanol	46	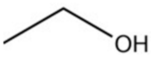	0.79	24.6
n-hexane	86	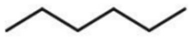	0.66	1.9
Isopropanol	60	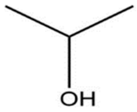	0.785	19.9

6. Comparison of Ra between the TpPa / PI membrane and COF membranes reported in the literature

Table S5 Comparison of *R*a between the TpPa / PI membrane and COF membranes reported in the literature

Name	Literature	*R*a（nm）
TpPa / PI membrane	This work	1.6
Tp-PaHz membrane	[1]	12.4
TpPPDTMC-50 / PAN membrane	[2]	12.1
TpBDTMC-25 / PAN membrane	[2]	14.5
PANI-TpPa / HPAN membrane	[3]	14.7

Reference

1. Zhu, Z.T.; Wang, H.J.; Cao, C.L.; Zou, J.Y.; Wang, M.D.; Zhang, Z.M.; Wang, Y.H.; Cao, Y.; Pan, F.S.; Chen, Y.; et al. Covalent organic framework membranes prepared via mixed linker modulated assembly for hydrogen peroxide enrichment. *Journal of Membrane Science* **2022**, *663*, 121043, doi:10.1016/j.memsci.2022.121043.
2. Zhao, S.; Zha, Z.Y.; Mao, C.Y.; Wang, Z.; Wang, J.X. In-situ fabricated covalent organic frameworks-polyamide hybrid membrane for highly efficient molecular separation. *Journal of Membrane Science* **2022**, *653*, 120544, doi:10.1016/j.memsci.2022.120544.
3. Mao, C.Y.; Zhao, S.; He, P.P.; Wang, Z.; Wang, J.X. Covalent organic framework membranes with limited channels filling through in-situ grown polyaniline for efficient dye nanofiltration. *Chem Eng J* **2021**, *414*, 128929, doi:10.1016/j.cej.2021.128929.
